# Retraction Note: Exosomal miR-4466 from nicotine-activated neutrophils promotes tumor cell stemness and metabolism in lung cancer metastasis

**DOI:** 10.1038/s41388-024-03269-w

**Published:** 2025-01-09

**Authors:** Abhishek Tyagi, Shih-Ying Wu, Sambad Sharma, Kerui Wu, Dan Zhao, Ravindra Deshpande, Ravi Singh, Wencheng Li, Umit Topaloglu, Jimmy Ruiz, Kounosuke Watabe

**Affiliations:** Department of Cancer Biology, Wake Forest University School of Medicine, Winston-Salem, NC 27157 USA

**Keywords:** Lung cancer, Metastasis, Tumour biomarkers, Inflammation

Retraction to: *Oncogene* 10.1038/s41388-022-02322-w, published online 23 April 2022

The authors have retracted this article. After publication, concerns were raised regarding highly similar ex vivo brain images in Figs. 2A (Vehicle, right), 5A (miR-4466 inh., left) and 6E (-Nic.+Stat3i, right). The authors thoroughly checked the underlying data and found that the data were mismanaged, which may have affected the presented results.

All authors agree with this retraction.

